# Secreted Neutrophil Gelatinase-Associated Lipocalin Shows Stronger Ability to Inhibit Cyst Enlargement of ADPKD Cells Compared with Nonsecreted Form

**DOI:** 10.3390/cells11030483

**Published:** 2022-01-30

**Authors:** Hsin-Yin Chuang, Wen-Yih Jeng, Ellian Wang, Si-Tse Jiang, Chen-Ming Hsu, Hsiu Mei Hsieh-Li, Yuan-Yow Chiou

**Affiliations:** 1 Department of Life Science, National Taiwan Normal University, Taipei 11677, Taiwan; grace870115@gmail.com (H.-Y.C.); barz1303@ntnu.edu.tw (C.-M.H.); 2 University Center for Bioscience and Biotechnology, National Cheng Kung University, Tainan 70101, Taiwan; wyjeng@mail.ncku.edu.tw; 3 Department of Biochemistry and Molecular Biology, National Cheng Kung University, Tainan 70101, Taiwan; 4 Division of Pediatric Nephrology, Department of Pediatrics, National Cheng Kung University Hospital, Tainan 70403, Taiwan; ellianwang@gmail.com; 5 Institute of Clinical Medicine, Medical College, National Cheng Kung University, Tainan 70101, Taiwan; stjiang@narlabs.org.tw; 6 National Laboratory Animal Center, National Applied Research Laboratories, Taipei 74147, Taiwan

**Keywords:** ADPKD, neutrophil gelatinase-associated lipocalin, 3D culture

## Abstract

Polycystic kidney disease (PKD) is one of the most common inherited diseases and is characterized by the development of fluid-filled cysts along multiple segments of the nephron. Autosomal dominant polycystic kidney disease (ADPKD) is the most common form of PKD, which is caused by mutations in either *PKD1* or *PKD2* genes that encode polycystin-1 (PC1) and polycystin-2 (PC2), respectively. As ADPKD progresses, cysts enlarge and disrupt normal kidney architecture, eventually leading to kidney failure. Our previous study showed that overexpression of exogenous kidney-specific neutrophil gelatinase-associated lipocalin (NGAL) reduced cyst progression and prolonged the lifespan of ADPKD mice (*Pkd1^L3/L3^*, 2L3 for short). In this study, we attempted to explore the underlying mechanism of reduced cyst progression in the presence of NGAL using immortalized 2L3 cells. The results of MTT and BrdU incorporation assays showed that recombinant mouse NGAL (mNGAL) protein significantly decreased the viability and proliferation of 2L3 cells. Flow cytometry and western blot analyses showed that mNGAL inhibited activation of the ERK and AKT pathways and induced apoptosis and autophagy in 2L3 cells. In addition, a 3D cell culture platform was established to identify cyst progression in 2L3 cells and showed that mNGAL significantly inhibited cyst enlargement in 2L3 cells. Overexpression of secreted mNGAL (pN + LS) and nonsecreted mNGAL (pN − LS) repressed cell proliferation and cyst enlargement in 2L3 cells and had effects on markers involved in proliferation, apoptosis, and autophagy. However, secreted mNGAL had a more pronounced and consistent effect than that of nonsecreted form. These results reveal that secreted mNGAL has stronger ability to inhibit cyst enlargement of ADPKD cells than that of nonsecreted form. These findings could help to identify strategies for the future clinical treatment of ADPKD.

## 1. Introduction

Polycystic kidney disease (PKD), one of the most common inherited diseases, is characterized by the development of fluid-filled cysts along multiple segments of the nephron [1]. ADPKD is the most common form of PKD, which is an adult-onset PKD for which most patients do not develop symptoms until they are in their forties [2]. ADPKD is the result of mutations in either *PKD1* (85%) or *PKD2* (15%) genes that encode polycystin-1 (PC1) and polycystin-2 (PC2), respectively [3,4]. Defects in PC1 or PC2 are associated with abnormal increases in the proliferation of renal epithelial cells and further cause cyst formation [5,6,7]. As ADPKD progresses, cysts enlarge and disrupt normal kidney architecture, eventually leading to kidney failure. However, the mechanism involved in ADPKD is complicated and remains to be explored. Functional loss of PC1 and/or PC2 in ADPKD leads to reduced intracellular calcium concentrations, alleviating calcium-sensitive adenylyl cyclase (AC) and further resulting in an increased level of intracellular cyclic adenosine monophosphate (cAMP) [8,9]. cAMP stimulates epithelial cell proliferation and fluid secretion to promote cyst enlargement by activating protein kinase A (PKA) and the downstream Ras/B-Raf/MEK/ERK pathway [10,11]. It has been demonstrated that the dysfunction of autophagy occurs in ADPKD zebrafish and mouse in vitro [12] and rat in vivo models [13]. Autophagy is a crucial mechanism that maintains cell and organ homeostasis by degrading and recycling organelles, long-living proteins and nutrients [14,15]. Cross-talk occurs between the mTOR pathway of autophagy and apoptosis pathways, which is dysregulated in PKD [16]. Treatment with autophagy activators, such as rapamycin and carbamazepine, can significantly attenuate cyst formation [12]. Previously, we established an ADPKD mouse model called *Pkd1^L3/L3^* (in a C57BL/6 genetic background) [17] by inserting a loxP site and a loxP-flanked mc1-neo cassette in introns 30 and 34 of the *Pkd1* gene, respectively (L3 means three loxP sites within the allele), to generate the conditional gene mutation. *Pkd1^L3/L3^* mice produced low levels (20% of the control) of PC1, which induced aggressive cyst growth and kidney volume expansion with increasing age [17]. In addition, an ADPKD cell model (2L3 cell line) was established by SV40 virus transduction of primary renal epithelial cells from *Pkd1^L3/L3^* mice to induce immortalized cell lines [18]. 

Neutrophil gelatinase-associated lipocalin (NGAL), a 22-kD secreted protein, is regarded as a biomarker for kidney injuries, including PKD in adults [19] (but not in children [20]), chronic kidney disease [21] and acute kidney ischemia [22]. The mechanism of NGAL protein increasing in patient urine is not clear, it has been proposed that accumulated NGAL in urine relates to deciliation of the renal epithelial cells as a result of injury [23]. Some studies have indicated that NGAL plays a protective role against renal injuries, amelioration of ischemia [24] and suppression of cyst progression [25], whereas some studies suggested NGAL might be involved in cyst progression [26] and tumor development [27]. NGAL receptor (NGAL-R, Slc22a17) is expressed in multiple epithelial tissues, including renal nephrons [28]. NGAL and NGAL-R coupling can sequester intracellular iron and further induce apoptosis [29], which is associated with an iron-depletion strategy of the innate immune system against bacterial infection [30]. 

Administration of exogenous NGAL has been shown to increase apoptosis of *Pkd1^−/−^* cells and further decrease cyst growth in vitro [25]. In previous work, we generated *Pkd1^L3/L3^* × *NGAL^−/−^* mice and *Pkd1^L3/L3^* × *NGAL^Tg/Tg^* mice by intercrossing *Pkd1^L3/L3^* mice with *NGAL* knockout and transgenic mice, respectively [31]. We found that overexpression of NGAL in an ADPKD mouse model (*Pkd1^L3/L3^* × *NGAL^Tg/Tg^* mice) reduced cyst progression and prolonged the lifespan of *Pkd1^L3/L3^* mice, accompanied by changes in a series of molecular pathways involved in proliferation, apoptosis and fibrosis [31]. However, the underlying mechanism of the effects of NGAL in alleviating the progression of ADPKD remains enigmatic.

In this study, a 3D cell culture platform was established to test the cyst progression of *Pkd1^L3/L3^* renal epithelial cells (2L3 cells) with the addition of recombinant mouse NGAL (mNGAL) protein or overexpression of transgenic NGAL in vitro. In addition, the effects of mNGAL on the proliferation, apoptosis, and autophagy-related pathways were explored. Furthermore, the mechanism of NGAL in 2L3 cells was elucidated by examining the effects of overexpression of secreted NGAL (pN + LS) and nonsecreted NGAL (pN − LS) in 2L3 cells. The effect and conceivable mechanism of NGAL protein in ADPKD cells were partially explored in this study.

## 2. Materials and Methods

### 2.1. Cell Lines

The *Pkd1^L3/L3^* (2L3) cell line was established by SV40 virus transduction of primary renal epithelial cells from 2L3 mice, an ADPKD mouse model, to induce an immortalized cell line [17,18]. Primary renal cells were infected in their second passage by using a Lenti-SV40 virus immortalization kit (Capital Biosciences Inc., Gaithersburg, MD, USA). The cells were incubated in 5% CO_2_ at 37 °C and treated with polybrene (8 µg/mL) overnight in a six-well plate. On the next day, the viral supernatant was discarded, and the cells were incubated in a fresh medium. After reaching confluence, the cells were subcultured into a 10-cm dish with culture medium and were considered passage one (P1) cells. SV40-transformed clones were selected, isolated by using cloning rings [17,18]. The M-1 cell line (*Pkd1*^+/+^) purchased from BCRC (Hsinchu, Taiwan) was used as the wild type cell line.

### 2.2. Cell Culture

The 2L3 cells were maintained in Dulbecco’s Modified Eagle Medium/nutrient mixture F-12 (DMEM/F12) containing 10% fetal bovine serum (FBS) and 1% penicillin/streptomycin (P/S) at 37 °C and 5% CO_2_. The cell culture reagents described above were purchased from Thermo (Waltham, MA, USA).

Cells were passaged every three days. To test the IC_50_ of mNGAL in ADPKD cells, 2L3 cells were seeded into 24-well plates (6000 cells/well) with 500 μL of the medium described above and incubated for 16 h. Cells were then treated with recombinant mNGAL (0, 20, 40, 80, 160, 320 μg/mL) or rapamycin (1 μM, from Sigma, St. Louis, MO, USA) for another 0, 8, 24, or 48 h (*n* = 4).

### 2.3. Lectin Staining

To check whether M-1 and 2L3 cells were from renal tubules of similar origins, lectin staining with *Lotus tetragonolobus* lectin (LTL, which is a differentiated proximal tubule marker labeled with fluorescein) and *Dolichos biflorus* agglutinin (DBA, which is a differentiated collecting duct marker labeled with rhodamine) was performed. LTL and DBA were purchased from Vector Laboratories (Burlingame, CA, USA).

For observation of cells, cells cultured on the coverslip were washed with phosphate-buffered saline (PBS) three times for 10 min and then fixed with 4% paraformaldehyde (PFA) in PBS for 30 min. Following permeabilization with 0.4% Triton X-100 in PBS (PBST) for 20 min, the cells were blocked with 10% horse serum in PBST for 1.5 h. The cells were stained with LTL (20 µg/mL) and DBA (20 µg/mL) for 1 h at 37 °C. Nuclei were stained with DAPI (1 μg/mL) for 5 min. Samples were washed with PBST three times for 10 min between the steps.

### 2.4. DNA Lysate and Genotyping

Cells were lysed with Direct-PCR lysis reagent (Viagen Biotech, Los Angeles, CA, USA) containing 0.4 mg/mL proteinase K (Bionovas, Canada) at 55 °C for 3 h, followed by 85 °C for 45 min to stop the reaction. The lysed product of genomic DNA was stored at −20 °C until PCR analysis. 

For *Pkd1* gene genotyping, PCR forward (5′-TGTGTTGTTCTTTGTGGCAGTCAG-3′) and reverse (5′-ATTCTCAATGACTGACTTGGGCTC-3′) primers were used to amplify genomic DNA sequences. Samples were heated at 94 °C for 5 min, and then 30 cycles (94 °C for 1 min, 56 °C for 1 min, 72 °C for 1 min) were performed followed by 72 °C for 10 min and 25 °C for 5 min. After amplifying by PCR, the DNA products were mixed with 6× loading dye and analyzed using 1% agarose Tris-borate-EDTA (TBE) gel.

### 2.5. Quantitative Reverse Transcription Real-Time Polymerase Chain Reaction (RT–qPCR)

Total RNA from the cell line was isolated using a MagQu gDNA extraction kit (MagQu, New Taipei City, Taiwan) followed by DNase I treatment (Worthington, Lakewood, NJ, USA) and stored in ddH_2_O at −80 °C. The quality and concentration of RNA were determined using a 1% TBE agarose gel and a NanoDrop spectrophotometer (Thermo Fisher Scientific). Template RNA was reverse transcribed to cDNA using a RevertAid First Strand cDNA synthesis kit (Thermo Fisher Scientific) in the presence of RNaseOUT^TM^ RNase inhibitor (G-Biosciences, St. Louis, MO, USA). Each reaction was prepared in a total volume of 20 μL, including total RNA (100 ng), Oligo (dT)_18_ primer (1 μL), 5X Reaction Buffer (4 μL), RiboLock RNase Inhibitor (20 U), dNTP Mix (1 mM), RevertAid Reverse Transcriptase (200 U), and nuclease-free water. For first-strand cDNA synthesis, samples were heated at 42 °C for 1 h, and the reaction was then terminated by heating at 70 °C for 1 min.

Quantification of gene expression was performed using a StepOne™ Real-Time PCR System (Applied Biosystems, Norwalk, CT, USA). Each sample was prepared in a total volume of 20 μL, including gene-specific forward (100 nM), reverse (100 nM) primers (Table 1), cDNA template (100 ng), ORA^TM^ SEE qPCR Green ROX H Mix (1×, highQu GmbH, Kraichtal, Germany) and PCR water. The samples were heated at 95 °C for 2 min, followed by 40 cycles (95 °C for 5 s, 65 °C for 25 s) and then melting curve analysis (95 °C for 15 s, 60 °C for 1 min, 95 °C for 15 s) using a StepOne™ Real-Time PCR System. Data were analyzed with StepOne™ Software (v2.3). Gene expression of *Gapdh* was used for normalization.

### 2.6. Cell Viability Assay

Cell viability was determined using thiazolyl blue tetrazolium bromide (MTT assay) (Sigma). MTT solution (0.5 mg/mL) was added to each well and incubated at 37 °C for 2 h. To solubilize the purple formazan crystals, dimethyl sulfoxide (DMSO, Sigma) was added to each well after removing the medium and MTT solution. The optical density (OD) of each sample at 570 nm was measured using an ELISA reader (Thermo Fisher Scientific).

### 2.7. Cell Proliferation Assay

The bromodeoxyuridine (BrdU) cell proliferation assay (Millipore, Burlington, MA, USA) was performed to analyze the proliferation of M-1 and 2L3 cells. To compare the proliferation ability of M-1 and 2L3 cells, cells were cultured in 96-well plates (5 × 10^3^ cells/well) for 24 h. BrdU was added to the medium 2 h prior to the end of the incubation period. To explore the effect of mNGAL on 2L3 cell proliferation, 2L3 cells were first cultured in 96-well plates (2 × 10^3^ cells/well) for 16 h followed by mNGAL treatment for another 8, 24, and 48 h. BrdU was added to the medium 2 h prior to the end of the mNGAL incubation period. Cells were then fixed with fixing solution (200 μL/well) at 37 °C for 30 min, followed by incubation with BrdU detection antibody (100 μL/well) at 37 °C for 1 h and peroxidase conjugated goat anti-mouse antibody (100 μL/well) at 37 °C for 30 min. The cells were washed three times with Wash Buffer and blotted dry on paper towels between the above-described steps.

After incubation with TMB peroxidase substrate (100 μL/well) at 37 °C for 30 min in the dark, the reaction was stopped with stop solution (100 μL/well). Finally, the OD_450_ of each sample was measured using an ELISA reader (Thermo Fisher Scientific).

### 2.8. Apoptosis Assay

For analysis of apoptosis using flow cytometry, 2L3 cells were seeded into 6-well plates (1.8 × 10^5^ cells/well) with 2 mL medium and incubated for 16 h. Cells were then treated with mNGAL (0, 600, 1200 μg/mL) for another 0, 4, or 12 h, collected and stained with Annexin V-FITC and PI (BD Biosciences, San Jose, CA, USA) for analysis by flow cytometry at 4 and 12 h, respectively (*n* = 3, for control and mNGAL). To establish compensation and quadrants before the analysis, unstained cells, cells stained with Annexin V-FITC only and cells stained with PI only were used.

### 2.9. Western Blot Analysis

Cells were harvested and lysed with RIPA supplemented with protease inhibitor cocktail (1:100) and phosphatase (1:100). Following incubation for 30 min at 4 °C, cell lysates were centrifuged for 15 min at 13,400× *g* (Eppendorf 5415R, Hamburg, Germany) at 4 °C. The concentrations of protein were measured using the BCA assay. Equal amounts of protein (25 μg protein with 1:2 loading dye/well) were electrophoretically separated by SDS–PAGE (10%) and transferred for 1 h to PVDF membranes. Membranes were then blocked with 5% skimmed milk in Tris-buffered saline with 0.1% Tween-20 (TBST) for 2 h at room temperature. After washing with TBST three times for 10 min, membranes were incubated with primary antibodies (Table 2) overnight at 4 °C, followed by a 1-h incubation with HRP-conjugated secondary antibodies (Table 3).

### 2.10. Three-Dimensional Cell Culture (3D Culture)

The 3D culture was performed using Matrigel matrix (Corning, NY, USA) to assess cyst formation. The 2L3 cells were seeded in 96-well plates (1000 cells/well) on preformed Matrigel (40% in medium). After the cells attached to the gel, more Matrigel was added to the cells to form a sandwich-like pattern (Figure 6). Cells were maintained in 3D culture at 37 °C and 5% CO_2_. Cells were treated with forskolin (20 mM, Sigma) from Day 1 to Day 3 to induce cyst formation. After the induction of cyst formation with forskolin, cells were treated with or without mNGAL (2 mg/mL) from Days 4 to 8, and the medium was changed every day. Cyst formation was observed under a microscope every day, and the cyst diameter, area, and number were measured with ImageJ. Immunocytochemistry (ICC) was performed at Day 8 on the plate or on coverslips. Observation of cysts was performed by confocal microscopy (ZEISS, Oberkochen, Germany).

### 2.11. Immunocytochemistry (ICC)

For observation of cysts, cysts cultured with Matrigel in wells or on coverslips were washed three times with PBS for 10 min and then fixed with 4% paraformaldehyde (PFA) in PBS for 30 min. Following permeabilization with 0.4% PBST for 20 min, cysts were blocked with 10% horse serum in PBST for 2 h. Cysts were stained with primary antibodies against E-cadherin (1:500; Cell Signaling Technology, Danvers, MA, USA) and actin (1:1000; Millipore) overnight at 4 °C and then stained with fluorescent secondary antibody (Table 3) in the dark for 1 h at room temperature. Nuclei were stained with DAPI (1 μg/mL) for 5 min. Samples were washed with PBST three times for 10 min between the steps.

### 2.12. Confocal Microscopy

To confirm that the spherical constructs observed in 3D culture were cysts, coverslips from 3D culture were mounted onto slides with Fluoromount-G mounting medium (SouthernBiotech, Birmingham, AL, USA) and imaged using a confocal microscope. Confocal imaging was performed with a 63× oil immersion objective. Different optical z sections of a 2L3 cyst in 3D culture were obtained using the Z-stack module in the ZEISS ZEN microscope software (Oberkochen, Germany). Optical sections of cysts were reconstructed into Z-stack or 3D video using ZEISS ZEN microscope software.

### 2.13. Plasmid Construction and Virus Infection

The mouse *NGAL* sequence (534 bp) was inserted into the pSecTag2B vector (Invitrogen, Carlsbad, CA, USA) in frame and downstream of a murine Igκ-chain leader sequence to overexpress the secreted NGAL protein. Conversely, the murine Igκ chain leader sequence in the above-described plasmid was removed to overexpress the nonsecreted NGAL protein. pSecTag2B-NGAL with or without the leader sequence (pN + LS or pN − LS, respectively) was designed to study the association between NGAL and the NGAL receptor. To ensure the correct insertion of constructs, *Nhe*I and *NotI* were used to digest pN + LS or pN − LS with NEBuffer 2 (New England Biolabs, Ipswich, MA, USA) at 37 °C for 2 h followed by analysis using a 1% TBE gel.

For infection of pN + LS and pN − LS into 2L3 cells, lentivirus particles of pN + LS and pN − LS were produced using the pLAS2.1w.PeGFP-I2-Bsd vector (from Academia Sinica, RNAi core, Taipei, Taiwan). pN + LS and pN − LS were subcloned into the pLAS2.1w.PeGFP-I2-Bsd vector from the pSecTag2B vector by digestion with *Nhe*I and *EcoR*I.

To generate stable clones, 2L3 cells were incubated in DMEM/F12 medium (300 μL/well) supplemented with 10% heat-inactive FBS, 1% P/S, polybrene (8 μg/mL) and lentivirus (200 μL) in a 6-well plate at 37 °C for 24 h. The virus supernatant was then discarded, and the cells were washed with PBS and incubated with fresh medium containing blasticidin (4 μg/mL) for selection of infected cells. The selection medium was changed every 3 days. Infected cells were harvested one week after blasticidin selection for western blotting and RT–qPCR to analyze the level of mNGAL expression.

### 2.14. Enzyme-Linked Immunosorbent Assay (ELISA)

Quantification of NGAL secreted by cells was performed using a mouse lipocalin-2 (Lcn2) solid-phase sandwich ELISA kit (Thermo Fisher Scientific). The supernatant medium of cells was collected, centrifuged to remove cell debris and stored at −80 °C. For the standard curve, 8× series dilutions of standard mouse NGAL protein were prepared (0, 0.024, 0.195, 1.563, 12.5, 100 ng/mL). Standards (100 μL/well) and samples (100 μL/well of 10 μg total protein from cell lysate or non-diluted supernatant) were added to the 96-well plate and incubated at 37 °C for 2.5 h. Each well was incubated with biotin (100 μL/well) conjugate at 37 °C for 1 h and then with streptavidin–HPR solution (100 μL/well) at 37 °C for 45 min with gentle shaking. The solution was discarded, and the well was washed with 1× Wash Buffer four times and blotted dry on paper towels between the above-described steps.

After incubation with TMB substrate (100 μL/well) at 37 °C for 30 min in the dark, the reaction was stopped by addition of stop solution (50 μL/well). Finally, the OD_450_ of each sample was measured using an ELISA reader (Thermo Fisher Scientific).

### 2.15. Statistical Analysis

All data are shown as the mean ± standard deviation (SD). For multiple mean comparisons, data were analyzed using the Student’s *t*-test and one-way analysis of variance (ANOVA) with Tukey’s post hoc tests in R studio. Differences were considered statistically significant when *p* value < 0.05.

## 3. Results

### 3.1. Comparison of NGAL Expression and Cell Proliferation between M-1 Wild Type and 2L3 ADPKD Cells

**Figure 1:**
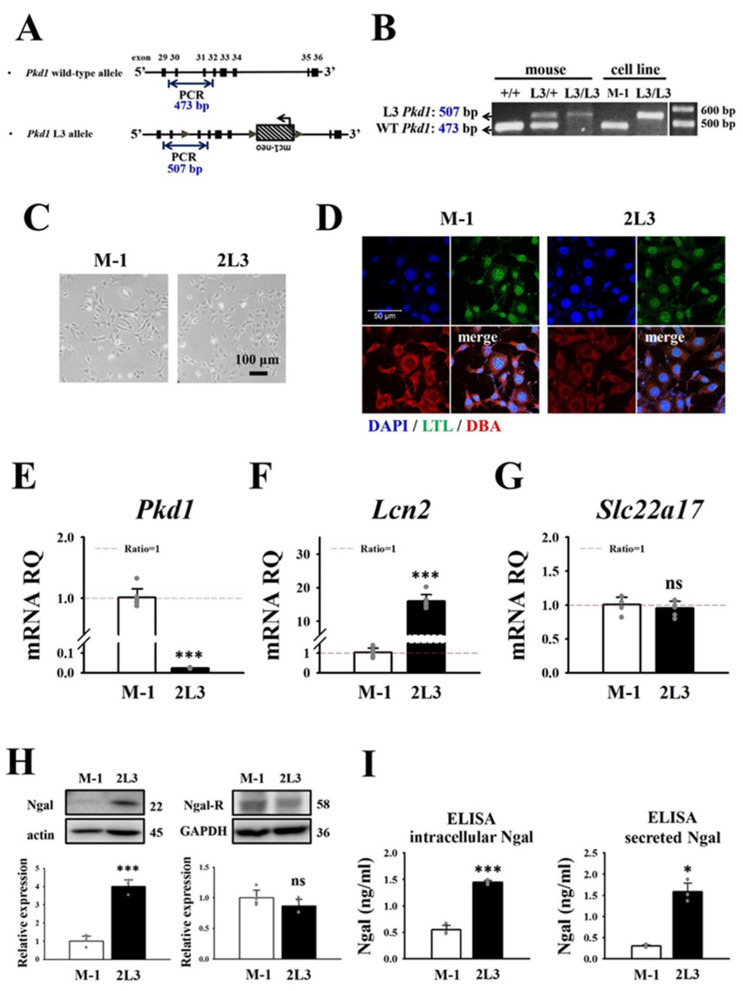
Comparison of NGAL expression between M-1 wild type and 2L3 ADPKD cells. (**A**) Structure of wild type and L3 alleles of Pkd1 gene. PCR genotyping was conducted using primers before and after the first loxP within the intron 30 of Pkd1. Filled triangles: loxP; an mc1 promoter-driven neomycin resistance gene (mc1-neo) flanked by two loxP sites was inserted into intron 34 of Pkd1. (**B**) PCR genotyping of Pkd1 transcripts of M-1 cells (473 bp) and 2L3 cells (507 bp). Pkd1 transcripts of the wild type (+/+), heterozygous (L3/+), and homozygous (L3/L3) mice were used as the control. RT–qPCR showed that 2L3 had significantly lower Pkd1 (**C**) M-1 and 2L3 cells show similar morphology. Scale bar: 100 μm. (**D**) M-1 and 2L3 cells were stained with Lotus Tetragonolobus Lectin (LTL, labeled with Fluorescein, green) and Dolichos biflorus agglutinin (DBA, labeled with Rhodamine, red) and DAPI (blue) and observed by a confocal microscope. Scale bar: 50 μm. RT–qPCR showed that 2L3 had significantly lower Pkd1 (**E**) and higher Lcn2 (NGAL) (**F**) expression levels than that of M-1 wild type cells. (**G**) No significant difference in Slc22a17 (NGAL-R) expression levels between M-1 and 2L3 cells. RQ: relative quantification = 2^−ΔΔCt^; Gapdh was used as the internal control of each sample. Values were normalized to M-1. (**H**) Western blot results showed that 2L3 cells had significantly higher expression of NGAL but not of NGAL-R than M-1 cells. Actin and GAPDH were used as the internal controls of each sample. Values were normalized to the control group of 2L3. (**I**) ELISA showed that 2L3 had higher expression of NGAL compared with M-1 in both cell lysate and supernatant. Student’s *t*-test was performed to determine the significance between the two groups (*n* = 6 in RT–qPCR, *n* = 3 in western blot and ELISA). * *p* < 0.05; *** *p* < 0.001 vs. M-1 of each group; ns: no significant difference.

The 2L3 cell line was established by SV40 virus transduction of primary renal epithelial cells from *Pkd1^L3/L3^* mice [17]. M-1 cells (mouse wild type renal epithelial cell line) purchased from BCRC (Hsinchu, Taiwan) were used as the control. PCR genotyping of *Pkd1* transcripts of 2L3 and M-1 cells was conducted to check the genotypes using primers before and after the first *loxP* site in intron 30 of the targeted allele (Figure 1A). *Pkd1*^+/+^, *Pkd1^L3/^*^+^ and *Pkd1^L3/L3^* mouse tissues were used as genotyping controls. PCR products from M-1 cells showed a single wild type (WT) *Pkd1*^+/+^ band (473 bp), while PCR products from 2L3 cells showed a single *Pkd1^L3/L3^* band (507 bp) due to an extra *loxP* segment (34 bp) (Figure 1B). 

To characterize whether M-1 and 2L3 cells were from renal tubules of similar origins, we first compared the morphology between the 2 cell lines; M-1 cells and 2L3 cells showed a very similar morphology (Figure 1C). In addition, lectin staining with *Lotus* tetragonolobus lectin (LTL, a marker of differentiated proximal tubules, labeled with fluorescein) and *Dolichos biflorus* agglutinin (DBA, a marker of differentiated distal tubules and collecting ducts, labeled with rhodamine) was performed to check their origins from renal tubules (Figure 1D). The staining results showed that M-1 and 2L3 cells were both LTL- (mostly colocalized with DAPI) and DBA-positive (localized in cytoplasm) (Figure 1D). This result suggested that M-1 and 2L3 cells originated from renal tubules of similar origins and might have similar characteristics.

Real-time PCR results showed that 2L3 cells had highly reduced *Pkd1* mRNA expression levels compared with M-1 cells (Figure 1E), which was consistent with the data from the 2L3 mice with *Pkd1* protein expression levels of only 25% of those of wild type mice [17]. The 2L3 mice have been demonstrated to have higher levels of NGAL and NGAL receptor (NGAL-R) protein in their kidneys than wild type mice [31]. Real-time PCR results showed that the *Lcn2* mRNA expression level was 15.96-fold greater in 2L3 than in M-1 cells (Figure 1F), consistent with data reported for 2L3 mice [31], while there was no significant difference in *Slc22a17* mRNA expression between the two groups (Figure 1G). Western blot results also showed that 2L3 cells had a higher expression level of NGAL than M-1 cells, while there was no significant difference in the expression level of NGAL-R between the two groups (Figure 1H). Cells and supernatants of M-1 and 2L3 cells were both harvested after culture for 48 h to quantify NGAL in the cell lysates and supernatants (representing the secreted NGAL) by ELISA. Consistent with the western blot results, ELISA showed that 2L3 had significantly higher intracellular NGAL and secretion of NGAL in the supernatant than M-1 (Figure 1I).

**Figure 2:**
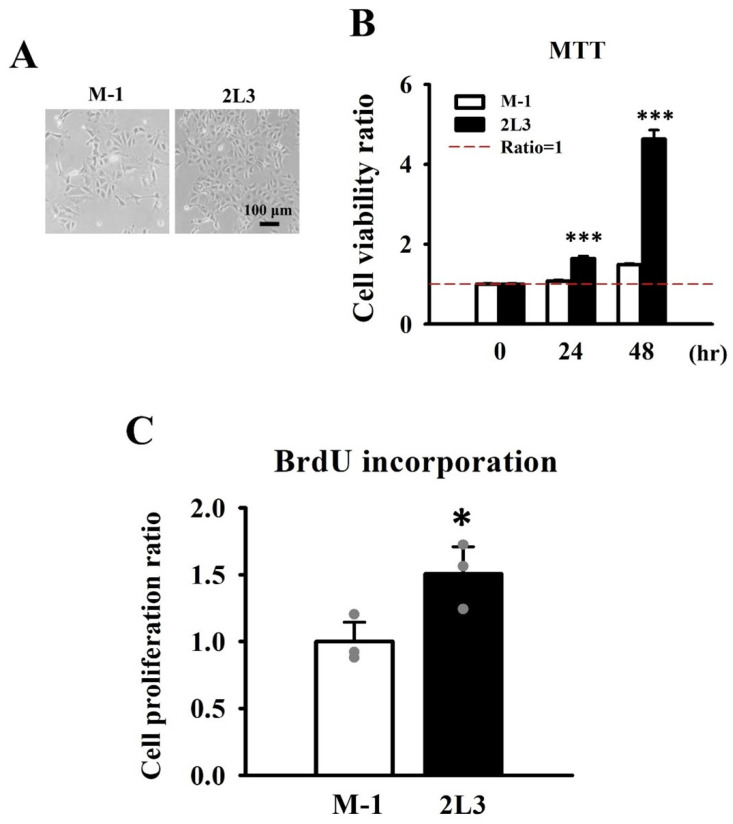
Comparison of cell viability and proliferation between M-1 wild type and 2L3 cells. (**A**) Representative images of M-1 and 2L3 cells cultured for 48 h. Scale bar: 100 μm. (**B**) MTT assay showed that 2L3 cells had a significantly higher viability than that of M-1 cells. (**C**) BrdU incorporation assay showed that 2L3 cells had a significantly better proliferation than that of M-1 cells. Values were normalized to M-1. Student’s *t*-test was performed to determine the significance between the two groups (*n* = 6 in MTT, *n* = 3 in BrdU incorporation). * *p* < 0.05; *** *p* < 0.001 vs. M-1 of each group.

The 2L3 mice have been demonstrated to show higher cell proliferation than wild type mice [17]. MTT and BrdU incorporation assays were conducted to investigate whether the 2L3 cell line also showed better viability and proliferation than wild type cells (Figure 2A–C). Representative images revealed greater confluence of 2L3 cells than M-1 cells after culture for 48 h with the same initial seeding numbers (Figure 2A). MTT assays showed that 2L3 cells had significantly better viability than M-1 cells cultured for 24 and 48 h (Figure 2B). To compare the proliferation ability of M-1 and 2L3 cells, the cells were cultured for 24 h following the BrdU incorporation assay, which showed that 2L3 cells had significantly better proliferation than M-1 cells (Figure 2C).

### 3.2. Effects of the Addition of mNGAL on the Viability and Proliferation of 2L3 Cells

**Figure 3:**
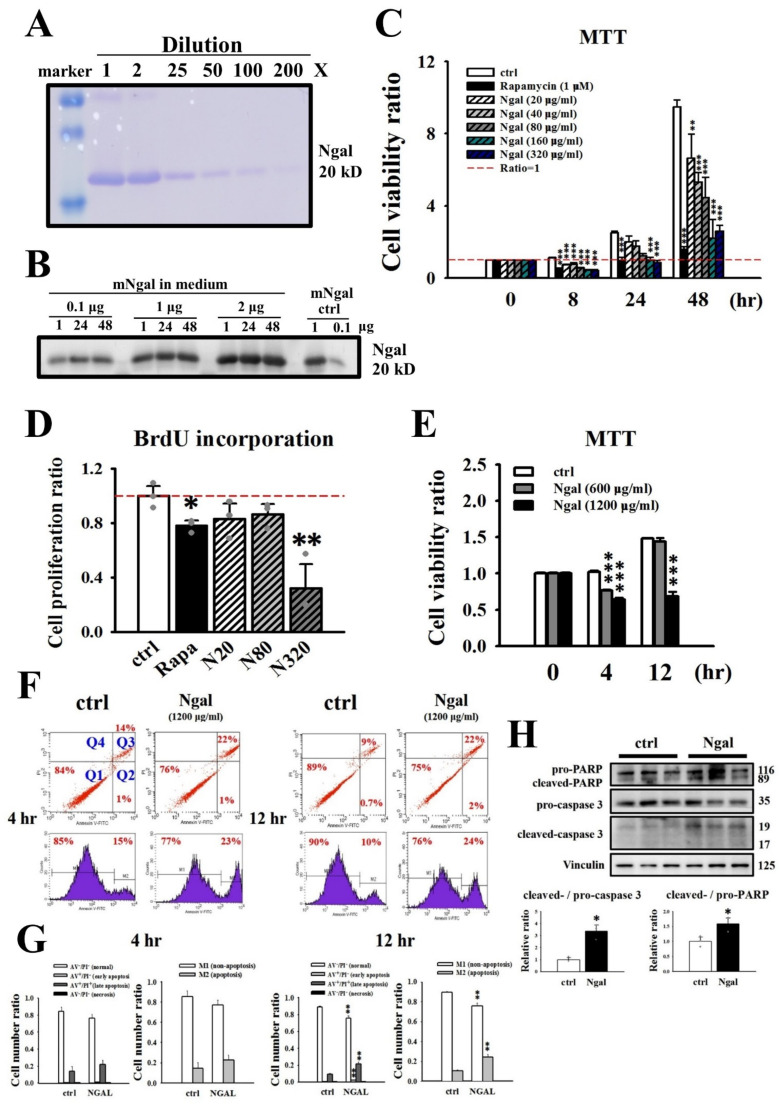
Effects of addition of mNGAL on cell viability and proliferation of 2L3 cells. (**A**) The result of SDS–PAGE showed that mNGAL (without signal peptide) purified from E. coli had a main 20-kD band and a light 40-kD dimer signals. (**B**) Western blot showed that mNGAL could be stably maintained in cell-culture medium for at least 48 h. (**C**) The result of MTT assay showed a concentration-dependent decrease in 2L3 cell proliferation following treatment with different concentrations of mNGAL (0, 20, 40, 80, 160, 320 μg/mL) on 8, 24, and 48 h in 24-well plates. Rapamycin was used as a positive control for proliferation inhibitor. (**D**) BrdU incorporation result showed that mNGAL (320 μg/mL) significantly reduced 2L3 cell proliferation on 24 h. Rapamycin was used as a positive control. (**E**) The result of MTT assay shows a decrease in 2L3 cell viability following treatment with different concentrations of mNGAL (0, 600, 1200 μg/mL) at 4 and 12 h in 24-well plates. (**F**) Cell apoptosis after 4 and 12 h treatment with mNGAL was detected using flow cytometry analysis using Annexin V (AV) and PI staining kit. Quadrantal diagram and histogram showed that 2L3 cells treated with mNGAL (1200 μg/mL) increased apoptosis at 12 h. The normal events (AV−/PI−) shown in lower right quadrant (Q1). The early apoptosis events (AV+/PI−) shown in lower right quadrant (Q2). The late apoptosis events (AV+/PI+) shown in lower right quadrant (Q3). The necrosis events (AV−/PI+) shown in lower right quadrant (Q4). The M-1 and M2 in the histogram represent cells that not undergoing apoptosis (AV−) and cells undergoing apoptosis (AV+), respectively. (**G**) Quantification of quadrantal diagram and histogram of 4 and 12 h, respectively. (**H**) Western blot results showed mNGAL treatment for 24 h increased apoptosis markers in 2L3 cells. Vinculin was used as the internal control. Values were normalized to the control group. One-way ANOVA or Student’s *t*-test was performed to determine the significance between groups (*n* = 3). * *p* < 0.05; ** *p* < 0.01; *** *p* < 0.001 vs. control of each group.

Administration of exogenous NGAL has been shown to decrease cyst growth in vitro [25]. Our previous study showed that overexpression of NGAL in an ADPKD mouse model (*Pkd1^L3/L3^*; *NGAL^Tg/Tg^* mice) reduced cyst progression accompanied by changes in a series of molecular pathways involved in cell proliferation, apoptosis and fibrosis [31]. Therefore, we investigated the effects of recombinant mNGAL on 2L3 cell viability using the MTT assay (Figure 3). The purity of recombinant mNGAL (without the signal peptide) purified from *E. coli* was first determined by SDS–PAGE and staining with Coomassie blue, which revealed a single 20-kD band and a very light dimer band at high concentrations (Figure 3A). In addition, the stability of mNGAL in the cell culture medium was analyzed by western blotting, which demonstrated the stability of mNGAL in medium for at least 48 h (Figure 3B). We noticed that mNGAL significantly decreased the viability of 2L3 cells compared with the control group after treatment for 8 h (Figure 3C). Rapamycin was used as a positive control in this experiment, which is recognized as an inhibitor of mTOR and is known to inhibit epithelial cell proliferation and cyst growth in a PKD mouse model [32]. In addition, the BrdU incorporation assay also showed that mNGAL treatment for 24 h significantly inhibited the proliferation of 2L3 cells (Figure 3D). Cell proliferation is a key feature of ADPKD, and the finding that 2L3 cell proliferation could be reduced by mNGAL suggested that mNGAL might further inhibit cyst formation in 2L3 cells.

### 3.3. Signaling Pathways Affected by the Addition of mNGAL to 2L3 Cells

NGAL and NGAL-R coupling has been shown to sequester intracellular iron and further induce apoptosis [29]. In addition, studies have shown that NGAL can induce apoptosis in an ADPKD model in vitro and in vivo [25,31]. To investigate whether mNGAL could induce apoptosis of 2L3 cells, we analyzed 2L3 cell apoptosis by flow cytometry. Based on the finding that mNGAL inhibited the proliferation of 2L3 cells after mNGAL treatment for 8 h (Figure 3D), we analyzed the effects of mNGAL on 2L3 cells at both earlier (4 h) and later (12 h) time points.

For flow cytometry analysis, much higher cell numbers and a shorter culture duration were required than those of previously conducted MTT assays; therefore, 2L3 cells were seeded onto 6-well plates, and a higher concentration of mNGAL (600–1200 μg/mL) was applied to the cells. The results showed that 1200 μg/mL mNGAL significantly inhibited cell viability after 4 and 12 h of treatment, while 600 μg/mL mNGAL inhibited cell viability only after 4 h of treatment (Figure 3E). Apoptosis assays were performed by flow cytometry to detect Annexin V-FITC- and PI-stained cells. Annexin V has a high affinity for phosphatidylserine (PS) and is used to identify apoptotic cells with exposed PS [33]. According to the staining results, cells can be classified as normal (AV−/PI−), early apoptosis (AV+/PI−), late apoptosis (AV+/PI+), and necrosis (AV−/PI+), and are shown in quadrantal diagrams (Figure 3F). Cells in the early apoptotic stage retain intact membranes, which differs from cells in the late apoptotic stage with disintegrated membranes [34]. The results showed that the 2L3 cell apoptosis rate (including early AV+/PI− and late AV^+^/PI^+^ apoptosis) was significantly increased compared with that of the control group after 12 h of treatment with mNGAL (1200 μg/mL; Figure 3F,G).

In addition, markers of apoptosis, cleaved forms of caspase-3 and poly (ADP-ribose) polymerase (PARP), were analyzed by western blotting. Caspase-3 is cleaved to the active form (17- and 19-kD fragments) through the two main pathways of apoptosis, extrinsic and intrinsic [35]. Cleaved caspase-3 sequentially cleaves PARP (89 and 24 kD fragments), a nuclear enzyme involved in DNA repair, to inactivate PARP and further induce apoptosis [35,36]. The western blot results (Figure 3H) were consistent with the flow cytometry data (Figure 3F,G). Treatment of 2L3 cells with mNGAL for 24 h significantly induced the ratio of cleaved/pro-caspase-3 and cleaved/pro-PARP (Figure 3H).

**Figure 4:**
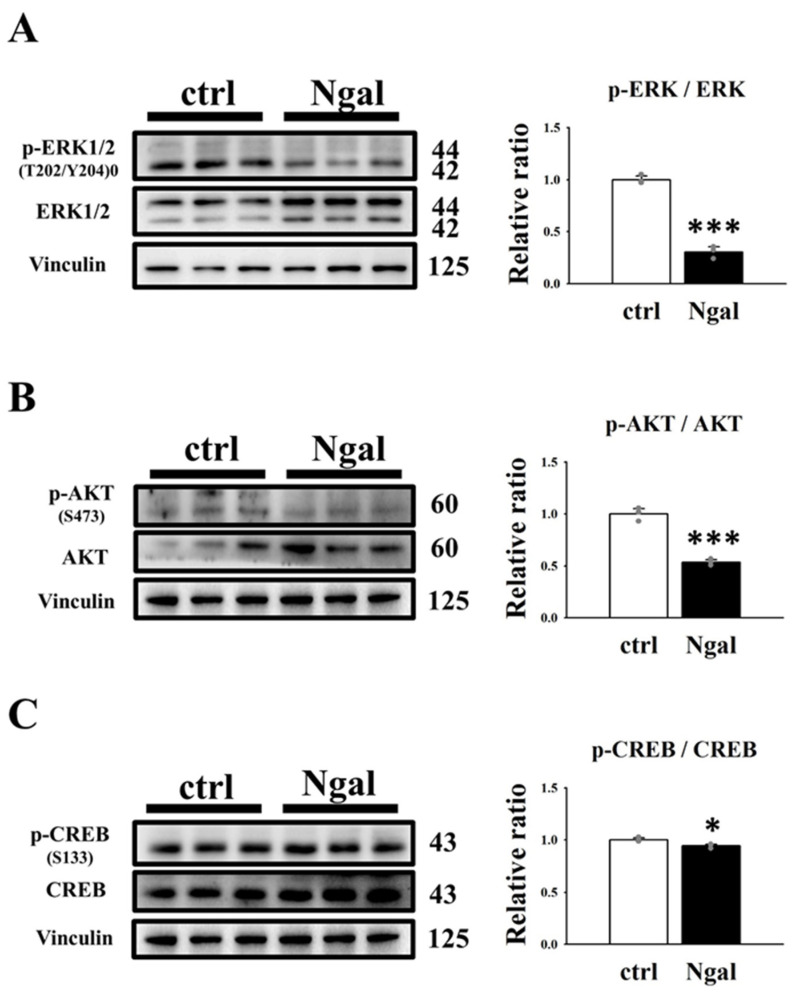
Effects of mNGAL on proliferation-related signaling pathways in 2L3 cells. Western blot results showed that mNGAL treatment for 24 h decreased the ratio of p-ERK/ERK (**A**) p-AKT/AKT (**B**) and p-CREB/CREB (**C**) in 2L3 cells. Vinculin was used as the internal control of each sample. Values were normalized to the control group. Student’s *t*-test was performed to determine the significance between groups (*n* = 3). * *p* < 0.05; *** *p* < 0.001 vs. control group.

**Figure 5:**
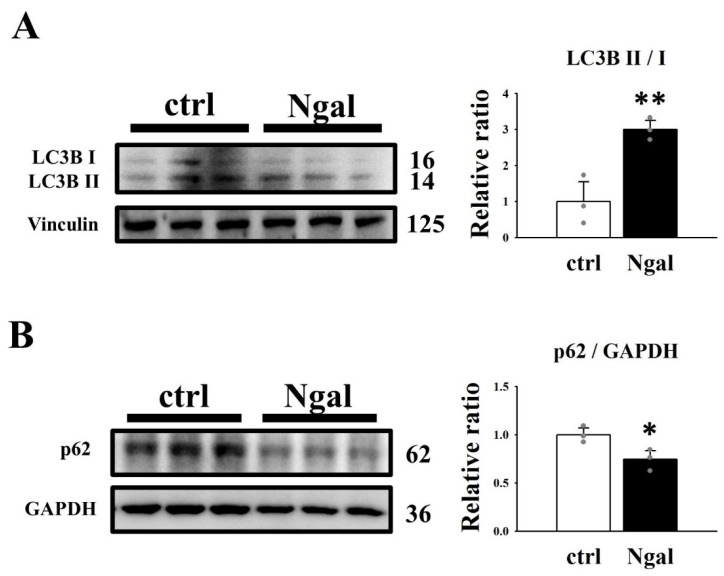
Effects of mNGAL on autophagy in 2L3 cells. Western blot results showed that mNGAL treatment for 24 h increased the autophagy-representing markers, LC3B II/LC3B I (**A**) and p62 (**B**) in 2L3 cells. Vinculin and GAPDH were used as the internal control of each sample. Values were normalized to the control group. Student’s *t*-test was performed to determine the significance between groups (*n* = 3). * *p* < 0.05; ** *p* < 0.01 vs. control group.

It has been demonstrated that the Ras/B-Raf/MEK/ERK [10,11] and AKT/mTOR [37,38] pathways are upregulated in ADPKD cells, which leads to increased cell proliferation and cyst enlargement. The MEK/ERK and PI3K/AKT signaling pathways lead to increased phosphorylation of cAMP-response element binding protein (CREB, S133) [39,40], a transcription factor that targets genes involved in cell survival and proliferation [41]. To investigate the mechanism by which mNGAL inhibits cell proliferation, western blotting was performed to analyze the effect of mNGAL on the ERK and AKT signaling pathways in 2L3 cells (Figure 4). The western blot results showed that mNGAL (1200 μg/mL) treatment of 2L3 cells for 24 h significantly reduced the ratio of p-ERK (T202/Y204)/ERK (Figure 4A), p-AKT (S473)/AKT (Figure 4B), and p-CREB (S133)/CREB (Figure 4C). These results (Figure 3 and Figure 4) showed that mNGAL could reduce cell viability of 2L3 cells through inhibiting cell proliferation and inducing apoptosis. It has been demonstrated that autophagy dysfunction occurs in ADPKD zebrafish and mouse models, while treatment with autophagy activators, such as rapamycin and carbamazepine can significantly attenuate cyst formation [12]. To investigate the effect of mNGAL on autophagy in 2L3 cells, two well-known autophagy markers, microtubule-associated proteins 1A/1B light chain 3 (LC3B) and p62, were analyzed by western blotting (Figure 5). The amount of LC3B II is correlated with autophagosome formation and is commonly used as a marker of autophagy [42]. The p62 protein directly links ubiquitin-tagged substrates and LC3B II and is eventually degraded, so the degradation of p62 is a widely used autophagy marker [43]. According to the western blot results, mNGAL treatment of 2L3 cells for 24 h significantly increased the ratio of LC3B II/LC3B I (Figure 5A) and reduced the level of p62 (Figure 5B) compared with the control group. This finding suggested that mNGAL activated autophagy in 2L3 cells.

### 3.4. Effects of mNGAL on Cyst Formation of 2L3 Cells in 3D Culture

**Figure 6:**
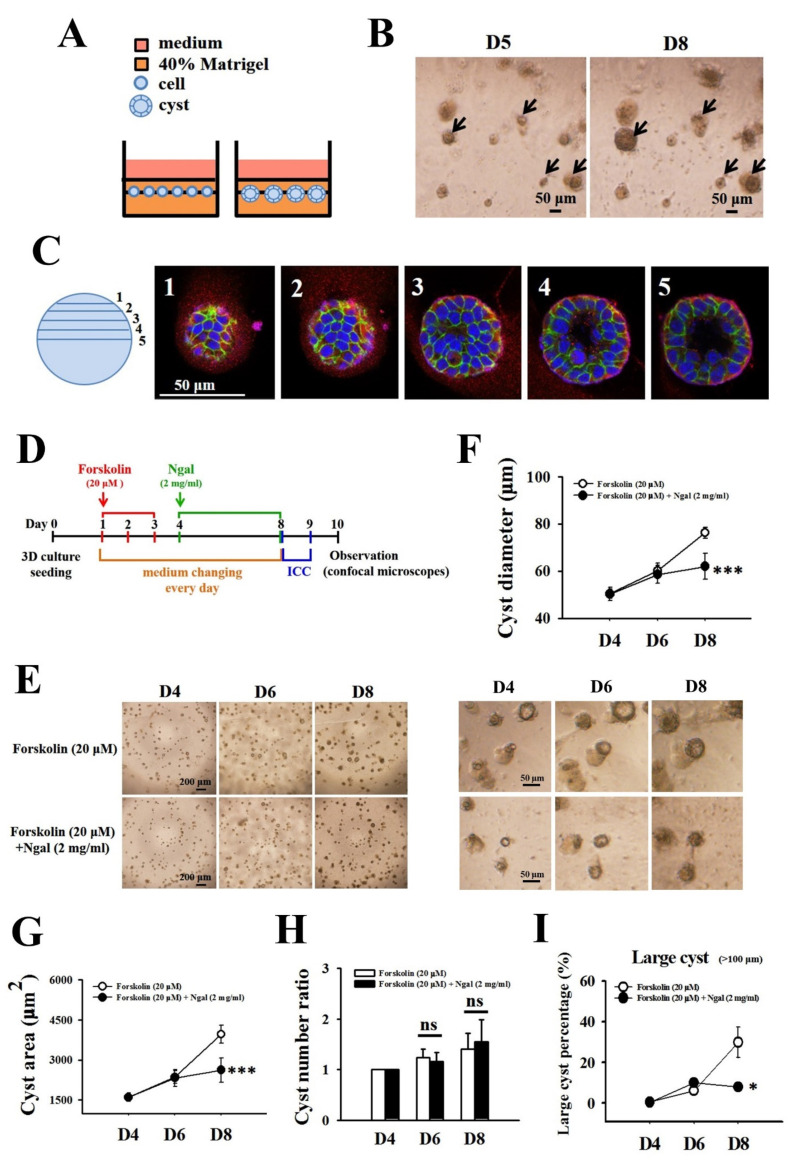
Effects of mNGAL on cyst formation of 2L3 cells in 3D culture. (**A**) Schematic diagram of the 3D culture performed in 96-well plate. The 2L3 cells were embedded in Matrigel (1000 cell/well) since Day 0, and medium changed twice a day. Formation of cysts which composed of many cells and a lumen was initiated approximately on Day 5. (**B**) Cyst morphology of 2L3 cells in 3D culture on Days 5 and 8 was observed by an optical microscope. Scale bar: 50 μm; arrows, cysts. (**C**) Different optical z sections of a 2L3 cyst in 3D culture from the top (layer 1) to the middle (layer 5). Cyst was fixed on Day 5 and stained with DAPI (blue) and E-cadherin (green) and actin (red) antibodies. Images were observed by a confocal microscope. Scale bar: 50 μm. (**D**) Timeline of 3D culture for 2L3 cyst formation. Cells were treated with forskolin (20 mM) from Days 1 to 3 to induce cyst formation. Cells were then treated with or without mNGAL (2 mg/mL) from Days 4 to 8. Cyst formation was observed every day and cyst diameters, area and numbers were measured by ImageJ. ICC was performed at Day 8 in the plate or on the coverslips. Observation of cysts were performed by confocal microscope (ZEISS). (**E**) Representative images of 2L3 cyst formation treated with or without mNGAL (2 mg/mL) on Days 4, 6, and 8, respectively. Higher magnification images of two groups are shown in the right panel. Scale bar: 200 (left panel) and 50 (right panel) μm. (**F**) Measurement of cyst diameters during Days 4–8. (**G**) Measurement of cyst area during Days 4–8. Cyst area was framed at the middle layer of each cyst and determined by ImageJ (in control groups, *n* = 257, 301, and 330, respectively; in mNGAL groups, *n* = 280, 329, and 391, respectively). The increasing rate of cysts number (**H**) and percentage of large size of cysts (**I**) in 3D culture during Days 4–8. Cyst number on Day 4 of each group was used as the control to calculate cyst number ratio. Student’s *t*-test was performed to determine the significance between groups (*n* = 4). * *p* < 0.05; *** *p* < 0.001 vs. control group; ns: no significant difference.

Cell proliferation is abnormally increased in 2L3 mice and leads to cyst formation and enlargement [17]. Based on the previous results that mNGAL inhibited 2L3 cell proliferation in 2D culture (Figure 3), we speculated that mNGAL might further inhibit cyst formation in 2L3 cells. To investigate whether mNGAL could inhibit cyst formation in 2L3 cells, we established a 3D culture platform that provided an appropriate environment with Matrigel for cyst formation (Figure 6). Cells were seeded in 96-well plates (1000 cells/well) on top of preformed Matrigel overnight. After the cells attached to the gel, more Matrigel was added to the cells to form a sandwich-like pattern (Figure 6A). Cells were maintained in 3D culture at 37 °C and 5% CO_2_ for at least 5 days to form cysts. Cysts in 3D culture grew larger with a prolonged culture time (Figure 6B). To confirm that the spherical constructs observed in 3D culture were cysts with a lumen, we fixed and stained them with DAPI and E-cadherin and actin antibodies to identify cell nuclei, cell–cell adhesion and apical membrane, respectively. Different optical z sections of a 2L3 cyst in 3D culture from the top (layer 1) to the middle (layer 5) were observed by confocal microscopy, and the lumen was confirmed within the spherical cyst (Figure 6C, Supplementary material).

To investigate the effects of mNGAL on cyst formation of 2L3 cells in 3D culture, cells in 3D culture were treated with forskolin from Days 1 to 3 to induce cyst formation. The cells were then treated with or without mNGAL from Days 4 to 8, and cyst diameters were measured every day (Figure 6D). Cysts in 3D culture grew proportionally with the number of culture days, with diameters ranging from 40 to 120 μm (Figure 6E–G), while mNGAL significantly suppressed the cyst diameters (Figure 6F) and area (Figure 6G) until Day 8. However, there was no significant difference in the increase in cyst number between the control and mNGAL-treated groups from Days 4 to 8 (Figure 6H). We further analyzed the number of large cysts (diameter > 100 μm) between the two groups and found that mNGAL significantly reduced the percentage of large cysts compared with the control group on Day 8 (Figure 6I).

### 3.5. Effects of Transfection with pN + LS and pN − LS on NGAL Expression and Cell Proliferation in 2L3 Cells

To study whether the need for a higher concentration of mNGAL (2 mg/mL) in 3D culture to inhibit cyst growth of 2L3 cells (Figure 6) was due to the lack of posttranslational modification of mNGAL, we designed a construct for overexpression of mNGAL in 2L3 cells. In addition, it has been demonstrated that NGAL can interact with NGAL-R and further induce apoptosis [29]. Therefore, to understand whether NGAL interacted with NGAL-R to induce apoptosis and further inhibit cell viability(Figure 3) and cyst growth (Figure 6), a construct for overexpression of nonsecreted NGAL (without the leader sequence) was also transfected into 2L3 cells.

**Figure 7:**
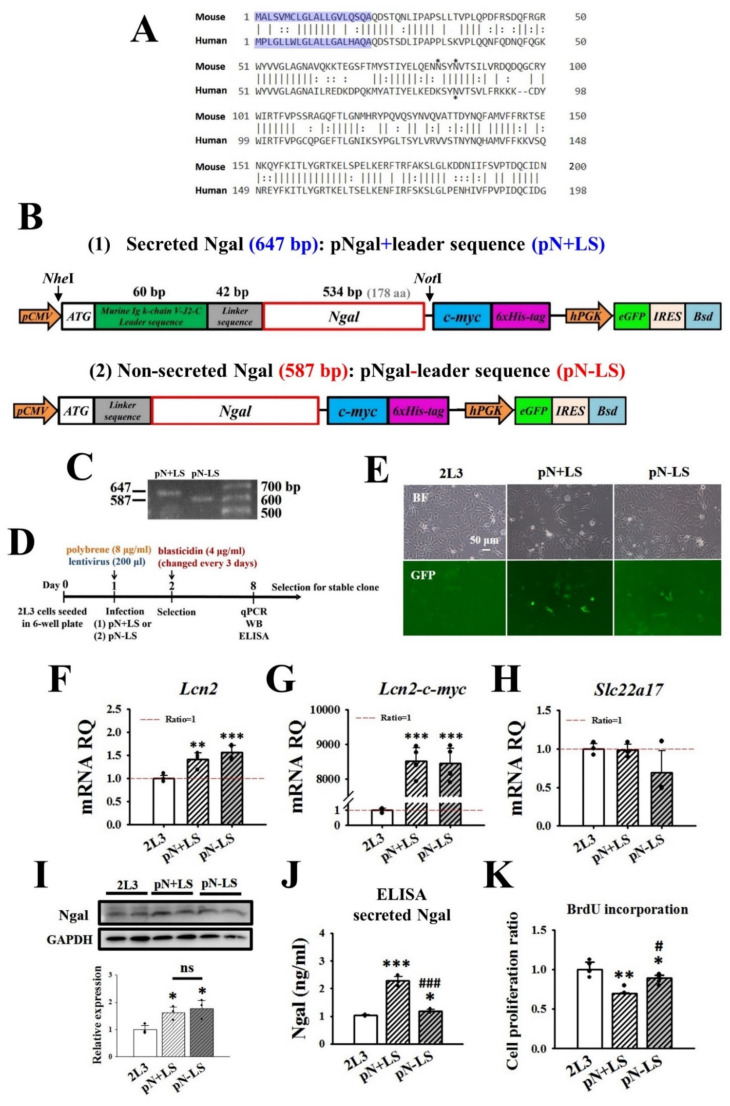
Effects of transfection with pN + LS and pN − LS on NGAL expression and cell proliferation in 2L3 cells. (**A**) Alignment of mouse NGAL (NP_032517.1) and human NGAL (NP_005555.2) proteins. The 20-aa signal peptides are shown in blue. The two reported N-linked glycosylation sites on Asn (N) at aa 81 and 85 of mouse NGAL and one at aa 85 of human NGAL are marked by asterisk (*). Identities: solid line; positives: paired dots. The sequence information above is from BLASTP program on National Center for Biotechnology Information (NCBI) website. (**B**) Construct diagram of mNGAL with (pN + LS) or without (pN − LS) the secreted Igκ leader sequence on the pLAS2.1w.PeGFP-I2-Bsd vector. (**C**) Result of gel electrophoresis confirmed the construct of pN + LS (647 bp) and pN − LS (587 bp) digested by *Nhe*I and *Not*I. (**D**) Timeline of infection and selection of 2L3 cells with pN + LS and pN − LS, respectively. The 2L3 cells were infected pN + LS or pN − LS in 6-well plate at Day 1. Selection with blasticidin started at Day 2 and medium was changed every 3 days. After selection for a week, cells were collected to be analyzed by RT–qPCR, western blot and ELISA. (**E**) Detection of expression of reporter eGFP to examine the transfection efficiency of pN + LS and pN − LS on Day 2. About 10% of cells were infected with the eGFP construct. Scale bar: 50 μm. RT–qPCR results showed that pN + LS and pN − LS both had higher expression of *Lcn2* (represented total NGAL level) (**F**) and *c-myc* (represented transfected exogenous NGAL level) (**G**) compared with the control group. (**H**) There was no significant difference of *Slc22a17* (NGAL-R) between the groups. RQ: relative quantification = 2^−ΔΔ^^Ϲ^^t^; *Gapdh* was used as the internal control of each sample. Values were normalized to the control group. (**I**) Western blot results showed pN + LS and pN − LS had higher expression of NGAL than the control group. (**J**) ELISA showed that pN + LS and pN − LS cells both secreted significantly higher NGAL than the control group, while the secreted NGAL amount of pN − LS cells was significantly less than pN + LS cells. (**K**) BrdU incorporation showed that pN + LS and pN − LS cells had lower cell proliferation ratio than the control group, while pN − LS cells had the higher of that than pN + LS cells. Student’s *t*-test was performed to determine the significance between the two groups (*n* = 3 in RT–qPCR and ELISA, *n* = 4 in western blot and BrdU incorporation). *p < 0.05; ** *p* < 0.01; *** *p* < 0.001 vs. 2L3 of each group; ^#^ *p* < 0.05; ^###^ *p* < 0.001 vs. pN + LS group; ns: no significant difference.

Mouse NGAL (NP_032517.1) and human NGAL (NP_005555.2) are 200-aa and 198-aa proteins, respectively, and both have a 20-aa signal peptide for secretion located at the N-terminus of the NGAL protein (Figure 7A). Sequence comparison showed 62% identity and 80% positivity between mouse NGAL and human NGAL. Mouse NGAL was shown to have two N-linked glycosylation sites on asparagine (Asn, N) at aa 81 and 85, while human NGAL had one site at aa 85 (Figure 7A), revealing the difference between them in posttranslational modification.

In this study, we used a murine Igκ-chain leader sequence (LS) [44] rather than the original signal peptide of mNGAL to ensure increased secretion of exogenous mNGAL protein. To overexpress secreted or nonsecreted NGAL, NGAL with or without LS in pLAS2.1w.PeGFP-I2-Bsd vectors, namely, pN + LS and pN − LS, were used and are shown in Figure 7B. Two promoters, *pCMV* and *hPGK*, were used to drive the independent expression of two proteins, NGAL-c-myc-His_6_ and eGFP. To ensure insertion of the construct, *Nhe*I and *NotI* were used to digest the two plasmids, and the gel electrophoresis results showed a 647-bp band from pN + LS and a 587-bp band from pN − LS (due to the lack of a 60-bp leader sequence) (Figure 7C).

For infection of pN + LS and pN-SP into 2L3 cells, polybrene and lentivirus were added to cells and incubated at 37 °C for 24 h, followed by selection with blasticidin (Figure 7D). Detection of GFP expression was performed by fluorescence microscopy after infection (Figure 7E). Infected cells were harvested one week after blasticidin selection for RT–qPCR, western blot and ELISA to analyze the level of NGAL expression (Figure 7F–J). The RT–qPCR results showed that infection of 2L3 cells with pN + LS and pN − LS led to increasing expression of *Lcn2* (Figure 7F) and *Lcn2-c-myc* (Figure 7G), which represented the levels of total and exogenous NGAL, respectively, while there were no differences in the levels of *Slc22a17* (NGAL-R) expression (Figure 7H). The western blot results showed that pN + LS and pN − LS cells had higher levels of intracellular NGAL expression than the control 2L3 cells (Figure 7I). ELISA results for the cell supernatant showed that pN + LS and pN − LS cells both had significantly higher levels of secreted NGAL than the 2L3 cell control group (Figure 7J). In addition, the amount of NGAL secreted by pN − LS cells was significantly less than that secreted by pN + LS cells (Figure 7J), while the two groups had similar NGAL expression levels (Figure 7I). This finding supported our prediction that overexpression of NGAL without a signal peptide (pN − LS) limited the secretion of NGAL despite the presence of a high level of intracellular NGAL. To investigate whether secreted NGAL rather than nonsecreted NGAL inhibited cell proliferation, a BrdU incorporation assay was performed, which showed that pN + LS and pN − LS cells had significantly lower proliferation than the control group (Figure 7K). In addition, pN − LS cells had significantly higher proliferation than pN + LS cells (Figure 7K). These results suggested that NGAL secretion from 2L3 cells was required to inhibit cell proliferation.

### 3.6. Effects of mNGAL Overexpression Leading to Signaling Pathways in 2L3 Cells

**Figure 8:**
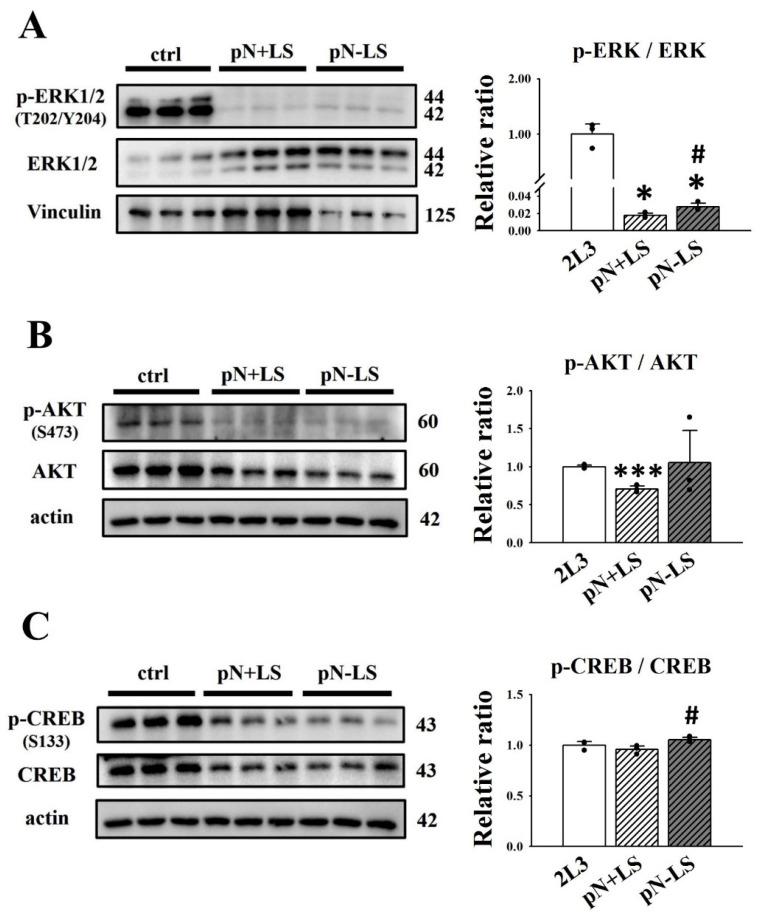
Effects of overexpression of mNGAL protein on proliferation-related signaling pathways in 2L3 cells. (**A**) Western blot results showed that pN + LS and pN − LS group had significantly reduced the ratio of p-ERK/ERK compared with the control group, and pN − LS group had the higher ratio than pN + LS. (**B**) pN + LS group had a lower ratio of p-AKT/AKT than the control group, while pN − LS cells had no significant difference compared to the control and pN + LS groups. (**C**) Ratio of p-CREB/CREB in pN − LS group was significantly higher than that of pN + LS group. Vinculin and actin were used as the internal control of each sample. Values were normalized to the control group. Student’s *t*-test was performed to determine the significance between groups (*n* = 3). * *p* < 0.05; *** *p* < 0.001 vs. control group; ^#^ *p* < 0.05 vs. pN + LS group.

Based on our previous results showing that mNGAL could inhibit the ERK and AKT pathways in 2L3 cells (Figure 4) and further decrease cell proliferation (Figure 3) and cyst enlargement (Figure 6), we used western blotting to analyze whether the ERK and AKT pathways were affected in pN + LS and pN − LS cells (Figure 8). The western blot results showed that the pN + LS and pN − LS groups had a significantly lower ratio of p-ERK (T202/Y204)/ERK than the control group, and the pN − LS group had a higher ratio of p-ERK (T202/Y204)/ERK than the pN + LS group (Figure 8A). The ratio of p-AKT (S473)/AKT was lower in the pN + LS group than in the control group, while pN − LS cells were not significantly different from those in the control and pN + LS groups (Figure 8B). The ratio of p-CREB (S133)/CREB was significantly higher in the pN − LS group than in the pN + LS group, while neither pN + LS nor pN − LS showed a significant difference from the control group (Figure 8C). These results suggested that pN + LS and pN − LS had statistically significant difference in ratio of p-ERK/ERK (Figure 8A), while pN + LS group did not show better effect in AKT (Figure 8B) and CREB (Figure 8C) activation.

**Figure 9:**
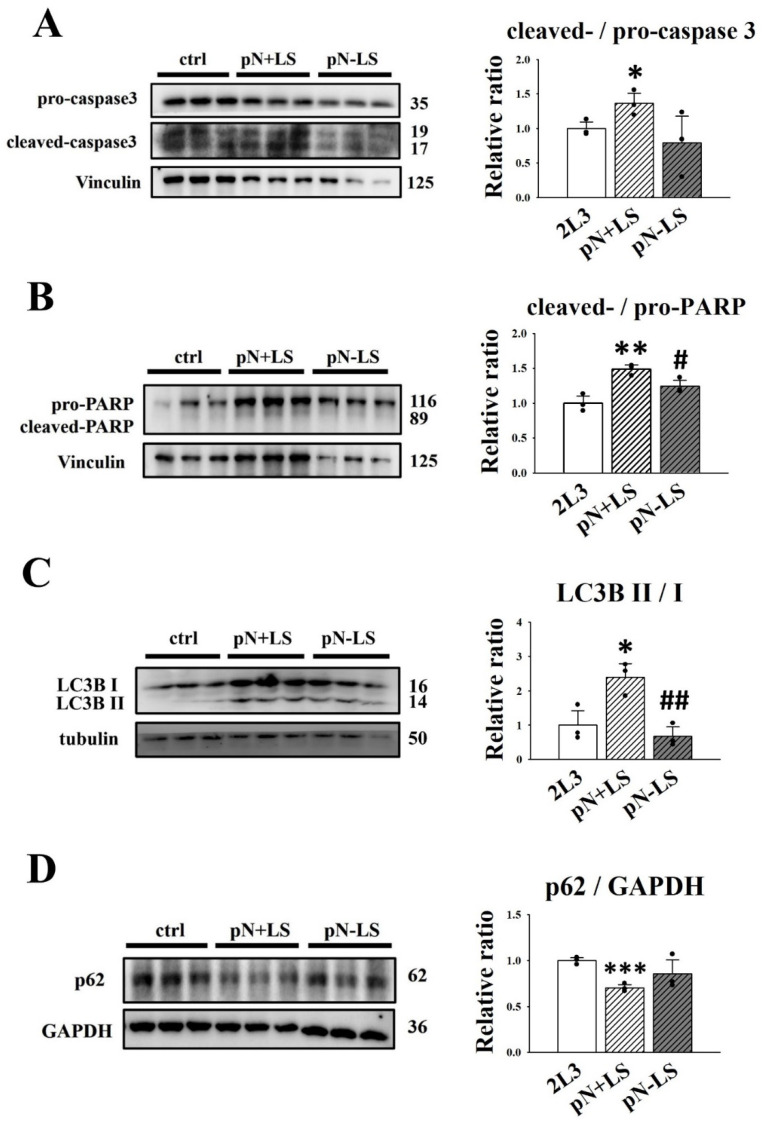
Effects of overexpression of mNGAL protein on apoptosis and autophagy in 2L3 cells. Western blot results showed that pN + LS cells had significantly higher ratio of cleaved-/pro-caspase-3 (**A**) and cleaved-/pro-PARP (**B**) than the control group, which represented the induction of apoptosis by secreted mNGAL. pN − LS cells had no significant difference in the ratio of cleaved-/pro-caspase-3 and cleaved-/pro-PARP compared with the control group. pN + LS cells had a higher ratio of LC3B II/LC3B I (**C**) and the lower ratio of p62 (**D**) compared with the control group. pN − LS cells had no significant difference in ratio of LC3B II/LC3B I and p62 compared with the control group. Tubulin, Vinculin, and GAPDH were used as the internal controls of each sample. Values were normalized to the control group. Student’s *t*-test was performed to determine the significance between groups (*n* = 3). * *p* < 0.05; ** *p* < 0.01; *** *p* < 0.001 vs. control group; ^#^ *p* < 0.05; ^##^ *p* < 0.01 vs. pN + LS group.

According to the previous results that mNGAL application could induce apoptosis (Figure 3) and autophagy in 2L3 cells (Figure 5) and decrease cell proliferation (Figure 3) and cyst enlargement (Figure 6), we used western blotting to investigate the effect on apoptosis and autophagy in pN + LS and pN − LS cells (Figure 9). The western blot results showed that pN + LS cells had a significantly higher ratio of cleaved-/pro-caspase-3 (Figure 9A) and cleaved-/pro-PARP (Figure 9B) than the control group; pN − LS cells showed no significant difference in the ratio of cleaved-/pro-caspase-3 (Figure 9A) and cleaved-/pro-PARP (Figure 9B) compared with the control group, and the ratio of cleaved-/pro-caspase-3 was significantly lower than that in the pN + LS group (Figure 9B). These results demonstrated that overexpression of secreted NGAL rather than nonsecreted NGAL induced apoptosis in 2L3 cells.

A higher ratio of LC3B II/LC3B I (Figure 9C) and a lower ratio of p62/GAPDH (Figure 9D) were identified in pN + LS cells compared with the control group; pN − LS cells had no significant difference in the ratio of LC3B II/LC3B I (Figure 9C) and p62/GAPDH (Figure 9D) compared with the control group, and the ratio of LC3B II/LC3B I was significantly lower than in the pN + LS group (Figure 9C). This result revealed that overexpression of secreted NGAL rather than nonsecreted NGAL induced autophagy in 2L3 cells.

### 3.7. Effects of Overexpression of mNGAL on Cyst Formation of 2L3 Cells in 3D Culture

Based on the previous results that mNGAL inhibited 2L3 cell proliferation and cyst enlargement in 2D and 3D culture, respectively (Figure 3 and Figure 6), we suspected that overexpression of secreted NGAL might further inhibit cyst enlargement of 2L3 cells through the inhibition of cell proliferation (Figure 7K).

**Figure 10:**
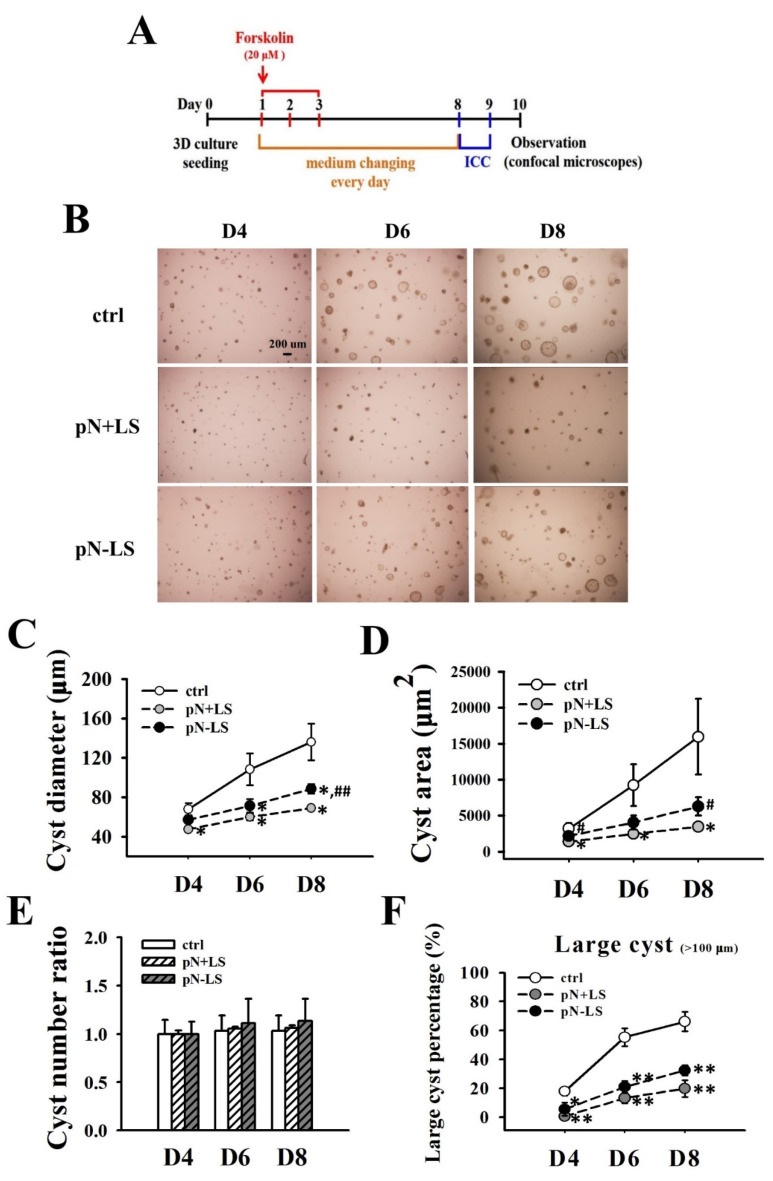
Effects of overexpression of mNGAL protein on cyst formation of 2L3 cells in 3D culture. (**A**) Timeline of cyst formation in 3D culture of the control group, pN + LS and pN − LS cells. (**B**) Representative images of cyst formation under the same view of the control group, pN + LS and pN − LS cells on Days 4, 6, and 8, respectively. Scale bar: 200 μm. (**C**) Measurement of cyst diameters of the control group, pN + LS and pN − LS cells during Days 4–8. (**D**) Measurement of cyst area of the control group, pN + LS and pN − LS cells during Days 4–8. Cyst area was framed at the middle layer of each cyst and determined by ImageJ (in control groups, *n* = 158, 163 and 163, respectively; in pN + LS groups, *n* = 142, 150 and 151, respectively; in pN − LS groups, *n* = 162, 180 and 184, respectively). (**E**) The cysts number in 3D culture of the control group, pN + LS and pN − LS cells during Days 4–8. Cyst number on Day 4 of each group was used as the control to calculate cyst number ratio. (**F**) Percentage of a large size of cysts in the 3D culture of the control group, pN + LS, and pN − LS cells during Days 4–8. Student’s *t*-test was performed to determine the significance between groups (*n* = 3). * *p* < 0.05; ** *p* < 0.01 vs. control group; ^#^ *p* < 0.05; ^##^ *p* < 0.01 vs. pN + LS group.

To investigate the effects of NGAL overexpression in 2L3 cells on cyst formation, the 2L3 control, pN + LS and pN − LS cells were treated with forskolin on Days 1–3 to induce cyst formation. The cyst diameters, area and numbers were measured daily after forskolin induction (Figure 10A). Cysts of the 2L3 control, pN + LS and pN − LS cells in 3D culture grew in a manner dependent on the culture days (Figure 10B) and showed obvious differences in cyst size from Days 4 to 8. The pN + LS group had significantly smaller cyst diameters (Figure 10C) and areas (Figure 10D) than the control group from Days 4 to 8, while the pN − LS group had significantly smaller cyst diameters than the control group on Days 6 and 8 (Figure 10C). There was no significant difference in cyst area between the control and pN − LS groups. In addition, the pN − LS group had significantly larger cyst diameters and areas than the pN + LS group on Day 8 (Figure 10C,D). Although there was no significant difference in the rate of cyst number increases among the control, pN + LS and pN − LS cells (Figure 10E), the pN + LS and pN − LS groups both had significantly lower percentages of large (diameter > 100 μm) cysts than the control group (Figure 10F) from Days 4 to 8. These results showed that pN + LS and pN − LS both inhibited cyst enlargement, while the inhibitory effect of pN − LS was weaker than that of pN + LS. This finding revealed that overexpression of secreted mNGAL had a superior ability to inhibit cyst enlargement of 2L3 cells in 3D culture than nonsecreted mNGAL.

## 4. Discussion

In this study, we demonstrated that the addition and/or overexpression of secreted mNGAL protein decreased 2L3 cell proliferation and inhibited cyst growth in vitro by upregulating apoptosis and autophagy and downregulating proliferation-related signaling pathways.

To identify whether M-1 cells, established from similar loci of mouse renal tubules as 2L3 cells, could be used as wild type control cells for 2L3 cells, lectin staining of M-1 and 2L3 cells with LTL and DBA was performed (Figure 1D). LTL and DBA were used to identify the differentiated proximal tubules and collecting ducts of mouse kidney, respectively [45,46]. The findings indicate that M-1 cells have similar morphology (Figure 1C) and origins of renal tubules (Figure 1D) to 2L3 cells; therefore, the M-1 cell line was selected as the wild type control for 2L3 cells.

In most clinical cases, ADPKD patients show higher levels of NGAL in their urine than normal people [19,47,48,49]. The ADPKD mouse model used in this study also has higher NGAL and NGAL-R expression levels [31]. Both RT–qPCR and western blot results showed that 2L3 cells expressed higher levels of NGAL than the M-1 wild type cell line (Figure 1F,H), whereas no significant difference in NGAL-R expression level was identified between the two cell lines (Figure 1G,H), which is inconsistent with the in vivo results [31]. This difference might be due to the complex interaction within the mouse tissues, which does not occur in the much simpler cell system.

The western blot results showed that direct administration and/or genetic overexpression of mNGAL in 2L3 inhibited the ERK pathway. As shown in Figure 4A and Figure 8A, the signal of p-ERK1 (44 kDa) was weaker than p-ERK2 (42 kDa), while ERK1 (44 kDa) was more intense than ERK2 (42 kDa), causing a marked increase in p-ERK2/ERK2 compared with p-ERK1/ERK1 in all experimental groups. ERK1 and ERK2 are two isoforms of ERKs that have been suggested to have different effects on cell proliferation [50]. ERK1 has been identified to induce cell survival and growth; however, ERK2 promotes cell differentiation and blocks cell growth [51]. In our study, the ratio of p-ERK2/ERK2 was notably higher than that of p-ERK1/ERK1 in all groups, which indicated that cells tended to differentiate and reduce growing. However, both the addition and overexpression of mNGAL dramatically lowered the ratio of both p-ERK1/ERK1 and p-ERK2/ERK2 compared with the control group and led to a significantly lower ratio of p-ERK/ERK, which indicating the mNGAL effect in inhibition of cell proliferation.

To establish a 3D culture platform for the characterization and treatment evaluation of cyst formation in 2L3 cells, several conditions were attempted. It has been demonstrated that the concentration of Matrigel for 3D culture influences the percentage of structural cysts and tubules formed in vitro [25,52,53]. In our study, we found that 40% Matrigel was the best formula to show the optimal cyst structure formation of 2L3 cells. There were several conditions, including embedded patterns and on-top patterns for 3D cultures [54]. We chose the on-top method (sandwich-like pattern) for a better and easier way to analyze the cysts (Figure 6). However, our experience showed that the cysts of 2L3 cells were not sufficiently large when cultured without any inducer in 3D culture until Day 14. Forskolin is commonly used to induce cyst formation by activating adenylyl cyclase (AC) to increase cyclic adenosine monophosphate (cAMP) [11,55]. Therefore, in our study, we used forskolin (20 μM) to induce cyst formation in 2L3 cells before mNGAL treatment. Our previous study showed that overexpression of kidney-specific NGAL in 2L3 mice could slow ADPKD progression by decreasing the cyst size but not the cyst number in 2L3 mice [31]. In the present study, the addition of mNGAL significantly inhibited cyst enlargement of 2L3 cells by suppressing the cyst diameter, area and the percentage of large cysts (Figure 6F,G,I), while there was no significant difference in the increasing rate of total cyst number (Figure 6H). The results of 2L3 cyst progression in 3D culture were consistent with the in vivo results. However, mNGAL treatment inhibited cyst growth of 2L3 cells in 3D culture, while we used a higher concentration of mNGAL (2 mg/mL) (Figure 6) than in the previous 2D experiments (600 μg/mL) for the MTT assay to examine the effect of mNGAL on cell viability (Figure 3). We speculated that recombinant mNGAL purified from *E. coli* lacked posttranslational modification [56,57] and resulted in a low bioactivity of recombinant mNGAL. Overexpression of NGAL in 2L3 cells by transfection of pN + LS and pN − LS constructs could help elucidate this question.

To determine whether a higher concentration of mNGAL (2 mg/mL) used in 3D culture to suppress cyst enlargement of 2L3 cells (Figure 6) was resulted from the lack of posttranslational modification of mNGAL and to test whether NGAL had to be secreted and to interact with NGAL-R to induce apoptosis and further inhibit cyst progression, pN + LS and pN − LS constructs were transfected into 2L3 cells to overexpress the secreted and nonsecreted NGAL proteins (Figure 7). Pooled transfected cells were harvested one week after blasticidin selection, both pN + LS and pN − LS transfection highly increased mRNA expression levels of *Lcn2* (total, including both endogenous and exogenous NGAL) and *Lcn2-c-myc* (exogenous NGAL) compared with the control group (Figure 7F,G), and there was no significant difference in *Lcn2* and *Lcn2-c-myc* between pN + LS and pN − LS groups (Figure 7F,G). The expression of *Lcn2-myc* in pN + LS and pN − LS groups was approximately 8500-fold greater than that in 2L3 cells (Figure 7G); however, the expression of *Lcn2* in pN + LS and pN − LS was approximately 1.5-fold greater than that in 2L3 cells (Figure 7F). In theory, *Lcn2-myc* was not present in 2L3 cells, leading to low expression levels (background) by RT–qPCR. As a result, the relative ratio of *Lcn2-myc* in pN + LS and pN − LS cells was markedly higher (approximately 8500 times) than that in the 2L3 control group, resulting in the large difference between the relative ratio of *Lcn2* and *Lcn2-myc*. In addition, the expression ratio of *Lcn2* in pN + LS and pN − LS cells (pN + LS was 0.91-fold that of pN − LS) was similar to that of *Lcn2-c-myc* (pN + LS was 1.01-fold that of pN − LS) (Figure 7F,G). Intracellular NGAL protein expression in pN + LS and pN − LS cells was increased compared with that in the control group (Figure 7I), and there was no significant difference between the pN + LS and pN − LS groups. The western blot results for total NGAL protein expression in the pN + LS and pN − LS groups (pN + LS was 0.92-fold that of pN − LS) (Figure 7I) were consistent with the RT–qPCR results (pN + LS was 0.91-fold that of pN − LS) (Figure 7F). According to these results, the levels of NGAL (both total and exogenous) proteins in pN + LS and pN − LS cells were similar, which indicated that the two groups should be compared in further experiments.

We predicted that overexpression of NGAL without a signal peptide would not increase NGAL secretion despite the high level of intracellular NGAL expression. However, the ELISA results using the supernatant from the culture showed that both pN + LS and pN − LS cells had significantly higher levels of secreted NGAL than the 2L3 control cells (Figure 7J), while the amount of NGAL secreted by pN − LS cells was significantly lower than that of pN + LS cells (Figure 7J). These results revealed that overexpression of NGAL without signal peptide (pN − LS) did limit the secretion of NGAL, although high levels of intracellular NGAL mRNA and protein were identified within the cells (Figure 7F,I). The BrdU incorporation results showed significantly higher proliferation of pN − LS cells than pN + LS cells (Figure 7K). 

The intracellular and secretion amounts of NGAL were approximately 1.6-fold and 2.2-fold higher in pN + LS than in 2L3 cells, respectively (Figure 7I,J), which could lead to the inhibition of pN + LS compared with 2L3 cell proliferation (Figure 7K). However, intracellular and secreted amounts of NGAL in 2L3 cells were approximately 4-fold and 5.3-fold higher than those in M-1 cells, respectively (Figure 1H,I), while higher cell proliferation was observed in 2L3 than in M-1 cells (Figure 2C). We suspect that increased NGAL expression and secretion, which play a protective role in 2L3 cells, is insufficient to inhibit the cell proliferation resulting from *Pkd1* knockout, but the increased NGAL expression and secretion in pN + LS provides supplemental NGAL to achieve proliferation suppression. In our previous study, *Pkd1^L3/L3^* mice had significantly greater renal levels of NGAL protein and proliferating cell nuclear antigen (PCNA) than *Pkd1*^+/+^ mice [31]. Nevertheless, the renal level of NGAL protein in *Pkd1^L3/L3^* × *NGAL^Tg/Tg^* mice was approximately 2-fold of that in *Pkd1^L3/L3^* × *NGAL*^+/+^ mice, and overexpression of NGAL led to markedly lower levels of PCNA in *Pkd1^L3/L3^* × *NGAL^Tg/Tg^* than in *Pkd1^L3/L3^* × *NGAL*^+/+^ mice and further inhibited cyst growth and prolonged survival days [31]. NGAL seems to play a protective role to inhibit the increased proliferation in ADPKD models, whereas endogenous NGAL expression is insufficient, and supplemental exogenous NGAL can help.

According to the 3D culture results (Figure 10), both the pN + LS and pN − LS groups inhibited cyst growth by lowering the diameter (Figure 10C), area (Figure 10D) and percentage of large cysts (diameter > 100 μm) (Figure 10F), but not the numbers (Figure 10E). This finding is inconsistent with our suspicion that only pN + LS but not pN − LS inhibits cyst enlargement because secretion of mNGAL from 2L3 cells is required for cell proliferation inhibition. However, inhibition of cyst enlargement by pN − LS was poorer than the pN + LS (Figure 10B–D), which revealed that overexpression of secreted mNGAL had a superior ability to inhibit cyst enlargement of 2L3 cells in 3D culture than that of nonsecreted mNGAL. Although we see stronger suppression of cyst enlargement in pN + LS group compared with pN − LS group in 3D culture, it is hard to see the better effect of pN + LS than pN − LS in p-AKT/AKT (Figure 8B), p-CREB/CREB (Figure 8C) and p62 (Figure 9D). It has been demonstrated that NGAL and NGAL-R coupling can sequester intracellular iron and further induce apoptosis [29], which is associated with an iron-depletion strategy of the innate immune system against bacterial infection [30]. We proposed that secreted mNGAL induced apoptosis in 2L3 cells through interacting with NGAL-R, whereas nonsecreted mNGAL could not interact with NGAL-R to induce apoptosis (Figure 9A,B). It has been demonstrated that the Ras/B-Raf/MEK/ERK [10,11] and AKT/mTOR [38] pathways are upregulated in ADPKD cells, which leads to increased cell proliferation and cyst enlargement. Our results showed that secreted mNGAL inhibited ERK (Figure 8A) and AKT (Figure 8B) pathways and nonsecreted mNGAL inactivated ERK pathway (Figure 8A). It suggests that mNGAL inactivates ERK pathway may not be necessary through NGAL-R. Knockout of NGAL-R in 2L3 cells will be performed to explore the question.

In addition, overexpression of mNGAL in 2L3 cells inhibited cell cyst enlargement (Figure 10) and occurred with a relatively lower secretion of NGAL (2.27 ng/mL) (Figure 7J) compared with the effect with high concentration of mNGAL (2 mg/mL) applied in previous 3D culture experiments (Figure 6). This result might be due to the lack of posttranslational modification of mNGAL from *E. coli* that was used in previous experiments, which required a high dose of mNGAL to inhibit cyst enlargement (Figure 6). To further examine whether a posttranslational modification of mNGAL is the factor responsible for the requirement of more recombinant mNGAL from *E. coli*, conditioned medium including mNGAL secreted from a mouse cell line will be used in further studies.

In the present study, pN + LS and pN − LS cells used for RT–qPCR, western blotting, ELISA, BrdU incorporation and 3D culture were obtained from a mixed polyclonal pool, which had varied transgene expression levels in the mixed population. Identification of the mechanism of mNGAL in ADPKD 2L3 cells by the above-described methods using clonal cells should aid in finding strategies for the future clinical treatment of ADPKD. 

## 5. Conclusions

In this study, we explored the underlying mechanism of reduced cyst progression in the presence of NGAL using an immortalized ADPKD cell line. The addition of recombinant mNGAL inhibited cell proliferation in ADPKD cells. A pathological analysis showed that mNGAL decreased proliferation and induced apoptosis and autophagy pathways in ADPKD cells. In addition, a 3D cell culture platform was established to identify cyst progression and showed that the addition of recombinant mNGAL inhibited cyst enlargement. Furthermore, overexpression of secreted mNGAL (pN + LS) had a stronger effect at inhibiting cyst enlargement of ADPKD cells than that of nonsecreted mNGAL (pN − LS), although both forms lowered the cyst diameter, the area and percentage of large size cysts, as well as their effects on proliferation, apoptosis, and autophagy. We conclude that secreted mNGAL has a more pronounced and consistent ability to inhibit cyst enlargement than that of nonsecreted mNGAL in ADPKD cells. Our work could help identify strategies for the future clinical treatment of ADPKD.

## Figures and Tables

**Table 1:** Primers used in real-time PCR of the cell lines.

Locus		Primer Sequence (5′-3′)	Product Size (bp)
*Gapdh*	Forward	CATCA CTGCC ACCCA GAAGA CTG	153
	Reverse	ATGCC AGTGA GCTTC CCGTT CAG	
*Lcn2*	Forward	CCACC ACGGA CTACA ACCAG	100
	Reverse	AGCTC CTTGG TTCTT CCATA CA	
*Lcn2-myc*	Forward	ATCTT CTCTG TCCCC ACCGA	98
	Reverse	ACGGC GCTAT TCAGA TCCTC	
*Pkd1*	Forward	TCAAT TGCTC CGGCC GCTG	102
	Reverse	CCAGC GTCTG AAGTA GGTTG TGGG	
*2Slc22a17*	Forward	CAGCC ACCTC CTAAC CGCTG TG	86
	Reverse	CTCCC ACTAG GCTCA AAGGC TGCT	

**Table 2:** Primary antibodies used in western blot (WB).

Antibody	Manufacture	Catalog	Titer	MW (kDa)	Host
NGAL	abcam	ab63929	1:500	20, 22	Rabbit
NGAL-R	NOVUS	NBP1-20975	1:1000	58	Goat
Caspase-3	Cell signaling	9662S	1:750	17, 19, 35	Rabbit
PARP	Cell signaling	9542S	1:1000	89, 116	Rabbit
p–ERK (T202/Y204)	Cell signaling	4377S	1:1000	42, 44	Rabbit
ERK	Cell signaling	9102S	1:1000	42, 44	Rabbit
p-AKT (S473)	Cell signaling	4060S	1:1000	60	Rabbit
AKT	Cell signaling	9272S	1:1000	60	Rabbit
p-CREB (S133)	Cell signaling	9198S	1:1000	43	Rabbit
CREB	Cell signaling	9197S	1:1000	43	Rabbit
LC3B	Cell signaling	2775S	1:1000	14, 16	Rabbit
p62	Millipore	MABC32	1:1000	62	Mouse
Actin	Millipore	MAB1501	1:5000	42	Mouse
GAPDH	Arigo	ARG10112	1:5000	36	Mouse
Vinculin	Millipore	05-386	1:2000	125	Mouse
tubulin	Thermo Fisher Scientific	A11126	1:2000	50	Mouse

**Table 3:** Secondary antibodies used in this study.

Antibody	Manufacture	Catalog	WB	ICC
Anti-mouse IgG HRP-linked	GE Healthcare	NA-931	1:2000	
Anti-rabbit IgG HRP-linked	GE Healthcare	NA-934	1:2000	
Anti-rabbit IgG 647	Invitrogen	A27040		1:200
Anti-mouse IgG 555	Invitrogen	A31570		1:200

## Data Availability

The data generated during the study are available from the corresponding author upon request.

## Supplementary Materials

Supplementary videos to this article can be found online (1) Video S1: Construction of a cyst in 3D culture: https://reurl.cc/Kjy8rq (accessed on accessed on 26 November 2021); (2) Video S2: Z-stack sections of a cyst in 3D culture: https://reurl.cc/VXGY5y (accessed on accessed on 26 November 2021). Supplementary graph: negative controls for Fig 1D (leaving out LTL and DBA staining) can be found online at https://reurl.cc/LpMmje (accessed on accessed on 26 November 2021).
